# In Vivo Trypanocidal Activity of the Drug Disulfiram Combined with Benznidazole

**DOI:** 10.1021/acsinfecdis.5c00936

**Published:** 2026-03-31

**Authors:** Viviane Flores Xavier, Kátia da Silva Fonseca, Thays Helena Chaves Duarte, Flávia de Souza Marques, Lucas Resende Dutra Sousa, Aline Tonhela Ferraz, Fernanda Caetano Camini, Daniela Caldeira Costa Calsavara, Cláudia Martins Carneiro, Brian Alejandro Suárez Mantilla, Ariel Mariano Silber, Paula Melo de Abreu Vieira

**Affiliations:** † Laboratory of Morphopathology, Department of Biological Sciences, Nucleus of Biological Sciences Research, Institute of Exact and Biological Sciences, 3057 Federal University of Ouro Preto, Ouro Preto, Minas Gerais 35402-133, Brazil; ‡ Laboratory of Immunopathology, Nucleus of Biological Sciences Research, Institute of Exact and Biological Sciences, Federal University of Ouro Preto, Ouro Preto, Minas Gerais 35402-133, Brazil; § Laboratory of Biochemistry Metabolic, Department of Biological Sciences, Institute of Exact and Biological Sciences, Federal University of Ouro Preto, Ouro Preto, Minas Gerais 35402-133, Brazil; ∥ Laboratory Biochemistry of Tryps, Department of Parasitology, Institute of Biomedical Sciences, University of São Paulo, São Paulo 05508-220, Brazil

**Keywords:** disulfiram, effectiveness, in vivo, trypanocidal activity, *Trypanosoma cruzi*

## Abstract

Chagas disease is a significant public health problem with benznidazole (BZ) and nifurtimox being the only available treatments. This study evaluated the trypanocidal activity of disulfiram (DS) in Swiss mice infected with the Y and VL-10 *Trypanosoma cruzi* strains. DS was tested alone or in combination with BZ at different doses for 20 consecutive days during the acute or chronic phase of the infection. Parasitological cure, inflammation in the heart and colon, and serum nitric oxide levels were assessed; in Y strain-infected animals, liver oxidative damage was also evaluated, while in VL-10-infected animals, parasitemia, survival rate, and collagen formation were analyzed. The combination of BZ + DS (100 mg/kg +50 mg/kg) induced 50% parasitological cure, reduced cardiac inflammation in Y strain infection, and decreased cardiac fibrosis in VL-10 strain infection. However, the results suggest a limited adjuvant effect of disulfiram, warranting further investigation.

## Graphical abstract

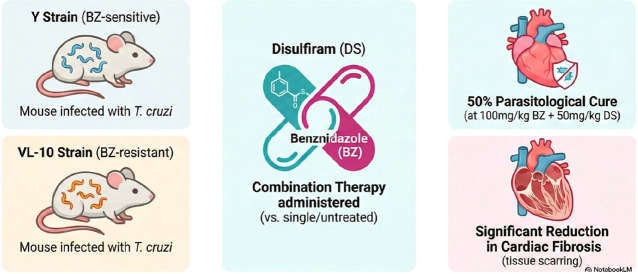

Chagas disease (ChD) was discovered in 1909 by the physician and scientist Carlos Chagas,^1^ and it is responsible for approximately 14000 deaths worldwide every year. ChD is currently endemic in 21 Latin American countries, and even though it is considered a major public health problem, less than 10% of people are diagnosed, with under 1% receiving the necessary treatment. Additionally, there are more than 7 million people infected with *Trypanosoma cruzi*, the etiological agent of the disease, with over 70 million people susceptible to the infection.^2,3^

During the course of the disease, two distinct clinical phases can be observed: the acute phase and the chronic phase. The acute phase begins shortly after infection and is characterized by patent parasitemia, intense tissue parasitism, as well as nonspecific signs and symptoms such as fever, malaise, lymphadenopathy, hepatosplenomegaly, and subcutaneous edema. In the cases of vector-borne transmission, Romaña’s sign and inoculation chagoma can be seen in infected patients.^4^ With the development of the immune response, parasitemia levels and parasite number in the tissues decrease substantially, thus characterizing the chronic phase of the disease.^5^

Approximately 60 to 70% of patients present the indeterminate form of the disease, characterized by positive anti *T. cruzi* serology, normal electrocardiogram and normal radiological examinations of the chest, esophagus, and colon. After remaining asymptomatic, about 30–40% progress to the chronic symptomatic phase is seen, characterized by the cardiac, digestive, or mixed form.^6,7^ The persistence of the parasite in the tissues during the chronic phase is directly and causally related to cell death, presence of inflammatory infiltrates, and reparative fibrosis.^8,9^

Regarding treatment, benznidazole (nitroimidazole) and nifurtimox (derived from nitrofuran) are the only available drugs,^2,10,11^ with both having significant trypanocidal activity in the acute phase. However, in the chronic phase, the efficacy is questionable.^12,13^ In addition, the effectiveness of these drugs can vary according to the different *T. cruzi* strains.^14,15^ Moreover, these drugs are frequently associated with significant adverse effects, including weight loss, nausea, vomiting, peripheral neuropathy, anorexia, and abdominal pain. About 30% of patients may also present changes in the central nervous system (CNS), such as polyneuritis, generalized or localized seizures, and even psychosis.^16^ Therefore, the limited efficacy of medications and frequent side effects are significant therapeutic obstacles as they lead to a decreased patient compliance with treatment.^13^

The lack of new and better treatment options is clearly an important point to be addressed.^17,18^ However, the search for new drugs requires investments in research and development as well as long study periods and significant financial resources. The repositioning of drugs that were first developed to treat other diseases has been presented as one of the fastest and most effective approaches for the introduction of new therapies.^19,20^ Another promising alternative is the association of drugs, which can allow dose reduction and hinder the emergence of resistance mechanisms.^21^

In this context, disulfiram (DS) (tetraethylthiuram disulfide), also commercially called antabuse or antiethanol, is a drug used in the treatment of chronic alcoholism and has been investigated for several other possible clinical applications.^22,23^ Data from the literature suggest a possible role for DS in the treatment of other types of addiction, such as Crack and Cocaine.^24^ DS demonstrated superior inhibition of bacterial growth in vitro compared to vancomycin in cases of bacterial infection, including vancomycin-resistant *Staphylococcus aureus* and vancomycin-resistant *Enterococcus faecium*.^25,26^ It was also effective against *Borrelia burgdorferi* in the stationary phase, with 99.8% inhibition of metabolic activity at a dose equivalent to 0.38 g/mL.^27^ DS has also shown effectiveness in the treatment of inflammatory conditions, neurological diseases, and cancers.^22,28–31^ Furthermore, it presents activities against parasites such as *Plasmodium falciparum*, *Giardia*, *Cryptosporidium parvum*, *Leishmania,* and *T. cruzi*.^22,32–35^

Diethyldithiocarbamate, the active metabolite of DS, has also demonstrated marked activity against *T. cruzi* in both in vitro and in vivo studies. Even at low concentrations, it was effective against amastigotes of the Y and Colombiana *T. cruzi* strains. When combined with BZ, diethyldithiocarbamate reduced epimastigote proliferation and increased the selectivity index by more than 10-fold. In in vivo models, cumulative survival of animals treated with the combination at a dose of 10 mg/kg/day (of each drug) increased approximately 6-fold. In the same study, the authors also evaluated the DS and BZ combination and observed a reduction in parasitemia in animals infected with the Y and Colombiana *T. cruzi* strains when compared with low doses of BZ (20/50 mg/kg).^36^

Other studies have also demonstrated that DS has a trypanocidal effect in epimastigotes (IC_50_ = 402 nM), with a delay in the logarithmic growth phase when compared to that in the control group. It was also observed that the amount of trypomastigote cells that hatched from the host cells in medium with the presence of DS decreased significantly.^33^ DS decreases the activity of the mitochondrial enzyme delta-1-pyrroline-5-carboxylate dehydrogenase (TcP5CDH) of *T. cruzi*, being this effect dose dependent with a reduction of enzyme activity (EC_50_) of 2.4 μM. TcP5CDH is the second enzyme in the proline oxidative pathway, which suggests that interference with proline metabolism would result in a decrease in the parasite’s viability through mechanisms that are still unclear.^37^ It is also important to highlight that a study on the safety and antitrypanosomal efficacy of DS in combination with BZ in chronic ChD is currently underway in Brazil.^22,38^

Thus, given the need to search for new drugs and the fact that DS can be a promising alternative, the goal of this work was to evaluate the trypanocidal activity of DS in Swiss mice infected with the Y and VL-10 *T. cruzi* strains. DS treatment in combination with BZ was able to promote parasitological improvement, resulting in the reduction of tissue injuries. However, the results were shown to be dose, strain, and tissue dependent.

## Results

### Y *T. cruzi* Strain

#### Evaluation of Parasitological Cures and Survival Rate

All animals in the untreated group and in the group treated with DS alone did not survive until the end of the acute-phase experiments with the Y *T. cruzi* strain. Animals that showed negative results of parasitism in the fresh blood examination (FBE) after immunosuppression and q-PCR (blood, heart, and colon) were considered cured. It is important to note that some animals died during the immunosuppression protocol; however, they had previously tested positive by FBE or blood q-PCR. Therefore, these animals were included in the final sample size for the calculation of the survival rate. The highest rate (50%) of parasitological cure in animals infected with the Y *T. cruzi* strain and treated in the acute phase was observed in the group treated with the BZ + DS (100 mg/kg +50 mg/kg), followed by cure rates of 33% and 10% observed in the groups treated with BZ at 100 mg/kg and 50 mg/kg, respectively (Table 1). No parasitological cure was observed in the remaining experimental groups.

**Table 1:** Parasitological Cure Data for Animals Infected with the Y *T. cruzi* Strain, Treated in the Acute Phase

			positives in q-PCR				
groups	survival (%)	FBE positive^a^ (%)	blood^b^ (%)	heart (%)	colon (%)	total positive animals (%)	cure (%)
BZ (100 mg/kg)	9/10 (90)	2/9 (22)	0/7 (0)	5/9 (55)	4/9 (44)	6/9 (67)	33
BZ (50 mg/kg)	10/10 (100)	8/10 (80)	0/2 (0)	5/7^e^ (71)	6/7^e^ (85	9/10 (90)	10
BZ + DS (100 mg/kg + 50 mg/kg)	10/10 (100)	4/10 (40)	1/6 (16)	0/6^f^ (0)	0/6^f^ (0)	5/10 (50)	50
BZ + DS (50 mg/kg + 50 mg/kg)	10/10 (100)	2/10 (20)	5/8 (62)	3/8^d^ (37)	7/8^d^ (87)	10/10 (100)	0
BZ + DS (100 mg/kg + 25 mg/kg)	7/10 (70)	5/7 (71)	1/2 (50)	3/6^c^ (50)	6/6^c^ (100)	7/7 (100)	0
BZ + DS (50 mg/kg + 25 mg/kg)	9/10 (90)	8/9 (88)	0/1 (0)	2/7^d^ (28)	7/7^d^ (100)	9/9 (100)	0

a Positive animals in the FBE after immunosuppression.

b Animals positive in q-PCR after immunosuppression and negative in the FBT.

c One animal died during the immunosuppression protocol.

d Two animals died during the immunosuppression protocol.

e Three animals died during the immunosuppression protocol.

f Four animals died during the immunosuppression protocol.

#### Inflammatory Process

All animals in the group infected with the Y *T. cruzi* strain and treated in the acute phase showed significantly higher inflammatory infiltrate in heart and colon tissues compared to noninfected (NI) animals (Figure 1). Interestingly, the cardiac muscle tissue of animals in the group treated with BZ (50 mg/kg) showed significantly higher inflammatory infiltrate when compared to the groups treated with BZ + DS (100 mg/kg +50 mg/kg) and BZ + DS (50 mg/kg +50 mg/kg) (Figure 1A). These findings are supported by photomicrographs showing varying degrees of inflammatory infiltrates among the treated groups (Figure 1B). Intense inflammation was observed primarily in animals treated with lower doses of BZ (50 mg/kg), while those receiving higher doses of BZ (100 mg/kg) or combination therapies (BZ + DS) independent of the doses exhibited moderate or lower infiltrates. In contrast, both the cardiac muscle tissue and the colonic muscle layer of noninfected (NI) animals maintained a normal histological appearance.

**Figure 1:**
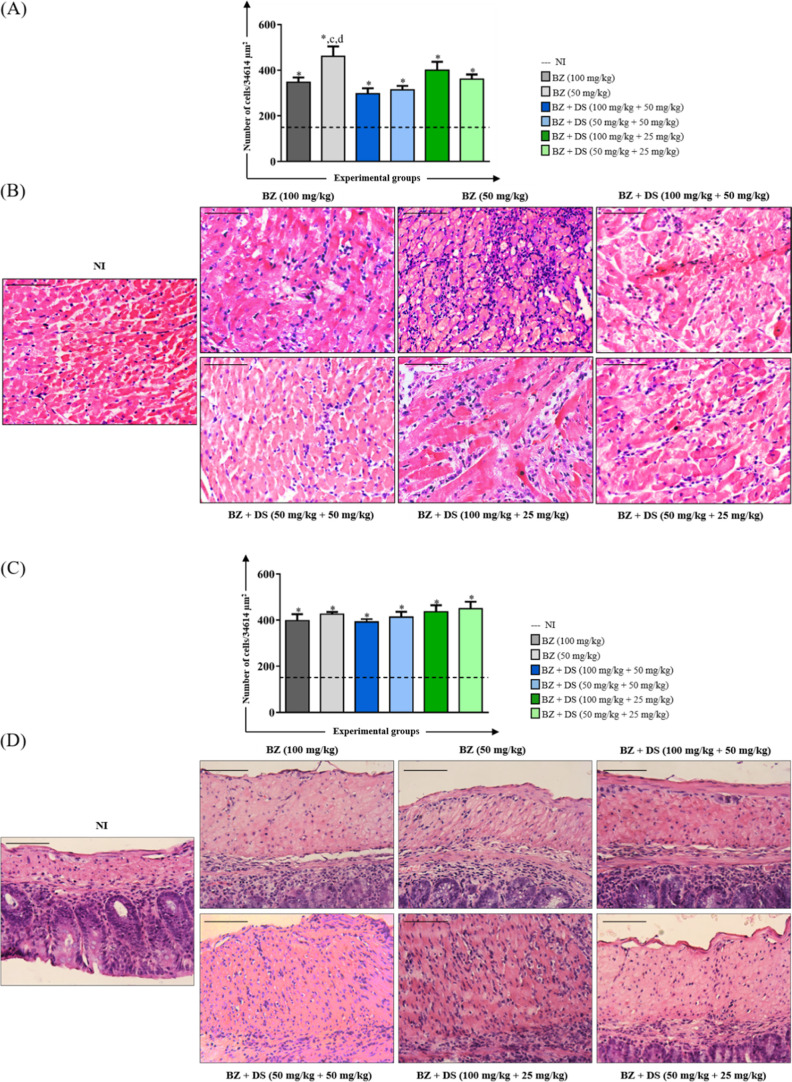
Morphometric analysis of the inflammatory process in the heart (A) and colon (C) and photomicrographs of histological sections of the heart (B) and colon (D) of mice infected with the Y *T. cruzi* strain and treated in the acute phase. * Number of inflammatory cells significantly higher (*p* < 0.05) than the uninfected group. The letters “c” and “d” represent significant differences (*p* < 0.05) in relation to the benznidazole (BZ) + DS (100 mg/kg +50 mg/kg) and BZ + DS (50 mg/kg +50 mg/kg) groups, respectively. The dashed line represents the average number of cell nuclei quantified in histological sections of uninfected animals. Values were expressed as mean ± standard error. Bar = 50 μm.

Regarding the analysis of the colon muscular layer, no significant differences were observed between the experimental groups that received treatment (Figure 1C). However, in the qualitative histological analysis, it was possible to observe an intense inflammatory infiltrate in the colon muscular layer of the animals in the BZ + DS (50 mg/kg +50 mg/kg) and BZ + DS (100 mg/kg +25 mg/kg) groups and moderate in the animals belonging to the other experimental groups (Figure 1D).

#### Quantification of Oxidative Damage in the Liver

Quantification of protein carbonyls and thiobarbituric acid reactive substances (TBARS) in the liver of animals infected with the Y *T. cruzi* strain (Figure 2) revealed that the combination of BZ + DS (100 + 25 mg/kg) resulted in significantly higher levels of oxidative damage markers compared with most other treatment regimens. Notably, this group and the BZ + DS group (50 mg/kg +25 mg/kg) presented significantly higher TBARS levels when compared to BZ monotherapy and higher–dose combination treatments, suggesting a dose-dependent effect of DS on oxidative damage.

**Figure 2:**
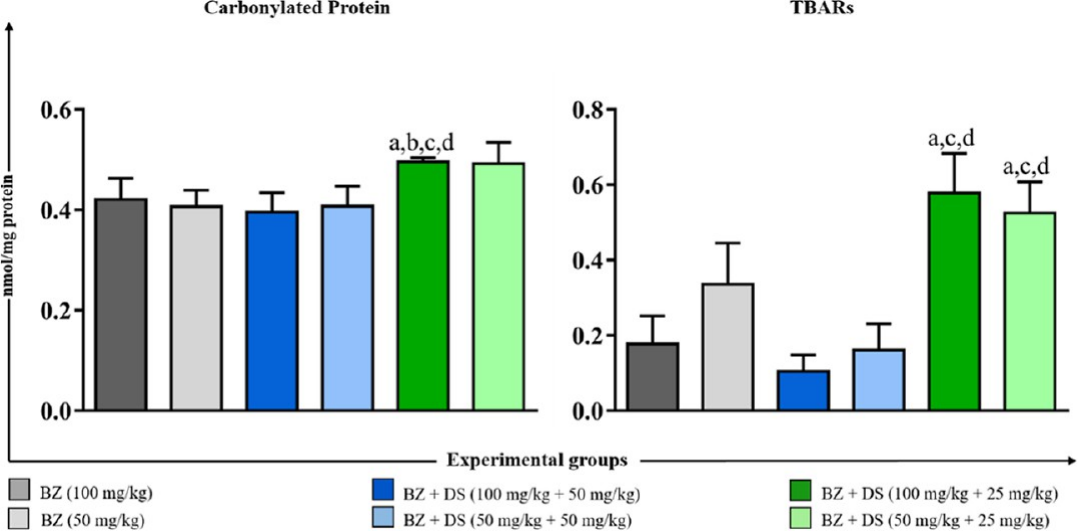
Quantification of protein carbonyl and TBARS in the liver of animals infected with the Y *T. cruzi* strain and treated in the acute phase. The letters “a”, “b”, “c” and “d” represent significant differences (*p* < 0.05) in relation to groups BZ (100 mg/kg), BZ (50 mg/kg), BZ + DS (100 mg/kg +50 mg/kg), and BZ + DS (50 mg/kg +50 mg/kg), respectively. Values were expressed as mean ± standard error.

### VL-10 *T. cruzi* Strain

#### Parasitemia

Figure 3 represents the results of parasitemia of animals infected with the VL-10 *T. cruzi* strain and treated in the acute phase with different drugs and schemes. As observed in Figure 3A, the infection was confirmed in 100% of the animals. The treatment of mice with the two dosages of DS (50 and 25 mg/kg) was not able to reduce parasitemia levels, which remained latent throughout this period. The analysis of the area under the parasitemia showed that the NT, DS (25 mg/kg) and DS (50 mg/kg) groups had an area significantly higher when compared to the other groups (Figure 3B). On the other hand, the association between BZ and DS, regardless of the used dose, showed important results of parasitemia control in the animals infected and treated.

**Figure 3:**
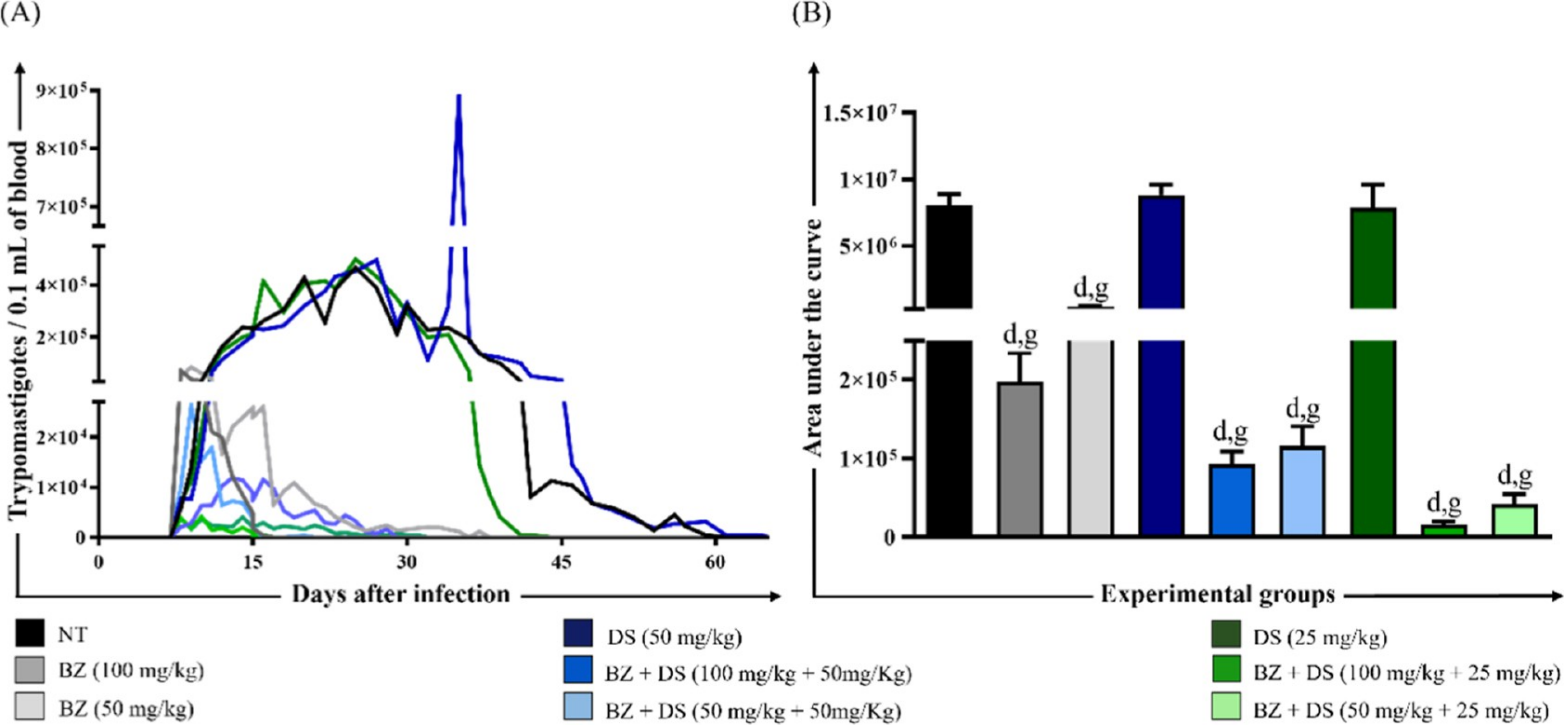
(A) Parasitemia of animals infected with the VL-10 *T. cruzi* strain and treated in the acute phase. The data are represented as an average of ten animals per group. NT: untreated; BZ: benznidazole; DS: disulfiram. (B) Area under the parasitemia of animals infected with the VL-10 *T. cruzi* strain and treated in the acute phase. The letters “d” and “g” represent significant differences (*p* < 0.05) in relation to groups DS (50 mg/kg) and DS (25 mg/kg), respectively. Values were expressed as mean ± standard error. NT: untreated.

#### Evaluation of Parasitological Cures and Survival Rate

The analysis of parasitological cure in animals infected with the VL-10 *T. cruzi* strain and treated in the acute phase showed that, regardless of the treatment (BZ, DS or combination), no cure was observed (Table 2). In addition, 100% of the animals showed FBT positivity in the acute phase.

**Table 2:** Parasitological Cure Data of Animals Infected with the VL-10 *T. cruzi* Strain, Treated in the Acute Phase

groups	survival (%)	FBE positive^a^ (%)	positives in q-PCR^b^ (%)	total positive animals (%)	cure (%)
NT	10/10 (100)	10/10 (100)	-	10/10 (100)	0
BZ (100 mg/kg)	10/10 (100)	10/10 (100)	-	10/10 (100)	0
BZ (50 mg/kg)	9/10 (90)	9/9 (100)	-	10/10 (100)	0
DS (50 mg/kg)	10/10 (100)	10/10 (100)	-	10/10 (100)	0
BZ + DS (100 mg/kg + 50 mg/kg)	10/10 (100)	10/10 (100)	-	10/10 (100)	0
BZ + DS (50 mg/kg + 50 mg/kg)	10/10 (100)	10/10 (100)	-	10/10 (100)	0
DS (25 mg/kg)	10/10 (100)	10/10 (100)	-	10/10 (100)	0
BZ + DS (100 mg/kg + 25 mg/kg)	9/10 (90)	9/9 (100)	-	10/10 (100)	0
BZ + DS (50 mg/kg + 25 mg/kg)	10/10 (100)	10/10 (100)	-	10/10 (100)	0

a Positive animals in the FBE after immunosuppression.

b Not performed.

In the chronic phase, survival rates among groups infected with the VL-10 *T. cruzi* strain ranged from 90 to 100% in the different treatment protocols (Table 3). Moreover, post-treatment parasitological evaluation of blood and other tissues showed no evidence of a cure in these infected animals.

**Table 3:** Parasitological Cure Data of Animals Infected with the VL-10 *T. cruzi* Strain, Treated in the Chronic Phase

			positives in q-PCR				
groups	survival (%)	FBE positive^a^ (%)	blood^b^ (%)	heart^c^ (%)	colon^c^ (%)	total positive animals (%)	cure (%)
NT	10/10 (100)	4/10 (40)	6/6 (100)			10/10 (100)	0
BZ (100 mg/kg)	10/10 (100)	5/10 (50)	3/5 (60)	2/2 (100)	2/2 (100)	10/10 (100)	0
BZ (50 mg/kg)	10/10 (100)	7/10 (70)	2/3 (66)	1/1 (100)	1/1 (100)	10/10 (100)	0
DS (50 mg/kg)	9/10 (90)	4/9 (44,4)	3/5 (60)	2/2 (100)	2/2 (100)	9/9 (100)	0
BZ + DS (100 mg/kg + 50 mg/kg)	10/10 (100)	6/10 (60)	1/4 (25)	3/3 (100)	3/3 (100)	10/10 (100)	0
BZ + DS (50 mg/kg + 50 mg/kg)	10/10 (100)	5/10 (50)	4/5 (80)	1/1 (100)	1/1 (100)	10/10 (100)	0
DS (25 mg/kg)	10/10 (100)	4/10 (100)	3/6 (50)	3/3 (100)	3/3 (100)	10/10 (100)	0
BZ + DS (100 mg/kg + 25 mg/kg)	10/10 (100)	7/10 (70)	0/3 (0)	3/3 (100)	3/3 (100)	10/10 (100)	0
BZ + DS (50 mg/kg + 25 mg/kg)	10/10 (100)	9/10 (90)	1/1 (100)			10/10 (100)	0

a Positive animals in the FBE after immunosuppression.

b Animals positive in q-PCR after immunosuppression and negative in the FBT.

c Negative animals in the q-PCR.

#### Inflammatory Process

The analysis of the inflammatory response in animals infected with the VL-10 *T. cruzi* strain and treated during the acute phase is shown in Figure 4. In the cardiac muscle tissue, treatment with BZ alone or in combination with DS led to a significant reduction in inflammatory infiltrates compared to the untreated group (Figure 4A). Furthermore, the group treated with BZ (50 mg/kg) presented significantly fewer inflammatory cells than those observed in animals treated with DS alone or with certain combinations of BZ + DS. These differences were also evidenced in the histological analysis (Figure 4B), which showed that animals treated with BZ (50 mg/kg) and BZ + DS (100 mg/kg +50 mg/kg) presented moderate inflammatory infiltrate, while animals belonging to the other experimental groups presented an intense one.

**Figure 4:**
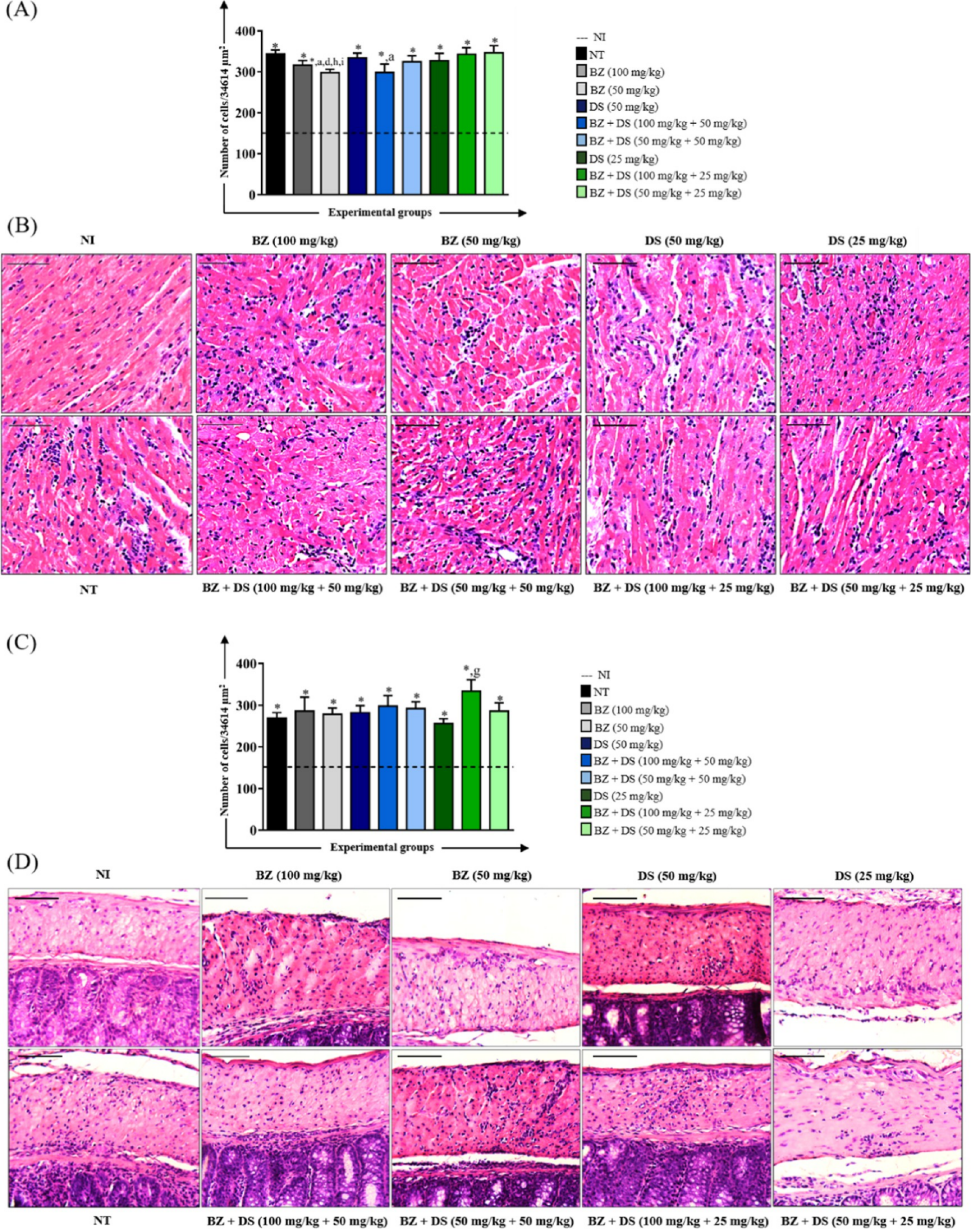
Morphometric analysis of the inflammatory process in the heart (A) and colon (C) and photomicrographs of histological sections of the heart (B) and colon (D) of mice infected with the VL-10 *T. cruzi* strain and treated in the acute phase. * Number of inflammatory cells significantly higher (*p* < 0.05) than the uninfected group. The letters “a”, “d”, “g”, “h” and “i” represent significant differences (*p* < 0.05) in relation to groups NT, DS (50 mg/kg), DS (25 mg/kg), BZ + DS (100 mg/kg +25 mg/kg), and BZ + DS (50 mg/kg +25 mg/kg), respectively. The dashed line represents the average number of cell nuclei quantified in histological sections of uninfected animals. Values were expressed as mean ± standard error. Bar = 50 μm.

In the analysis of the colonic muscle layer, the BZ + DS group (100 mg/kg +25 mg/kg) presented a significantly higher number of inflammatory cells than the NT and DS groups (25 mg/kg) (Figure 4C). The latter presented a discrete inflammatory infiltrate in the histological analysis, while the BZ + DS group (100 mg/kg +25 mg/kg) presented an intense one (Figure 4D). The other experimental groups did not present a significant difference in the statistical analysis as they exhibited only moderate inflammatory infiltrate in the histological analysis.

The quantitative analysis of the inflammatory process in the animals infected with the VL-10 *T. cruzi* strain and treated in the chronic phase is shown in Figure 5. In the cardiac muscle tissue, the NT group had a significantly higher number of inflammatory cells when compared with the BZ (100 mg/kg) and BZ + DS (100 mg/kg +25 mg/kg) groups (Figure 5A). Histological analysis confirmed that the experimental groups BZ (100 mg/kg) and BZ + DS (100 mg/kg +25 mg/kg) presented only a discrete inflammatory infiltrate, while the animals belonging to the other experimental groups presented moderate inflammatory infiltrate (Figure 5B).

**Figure 5:**
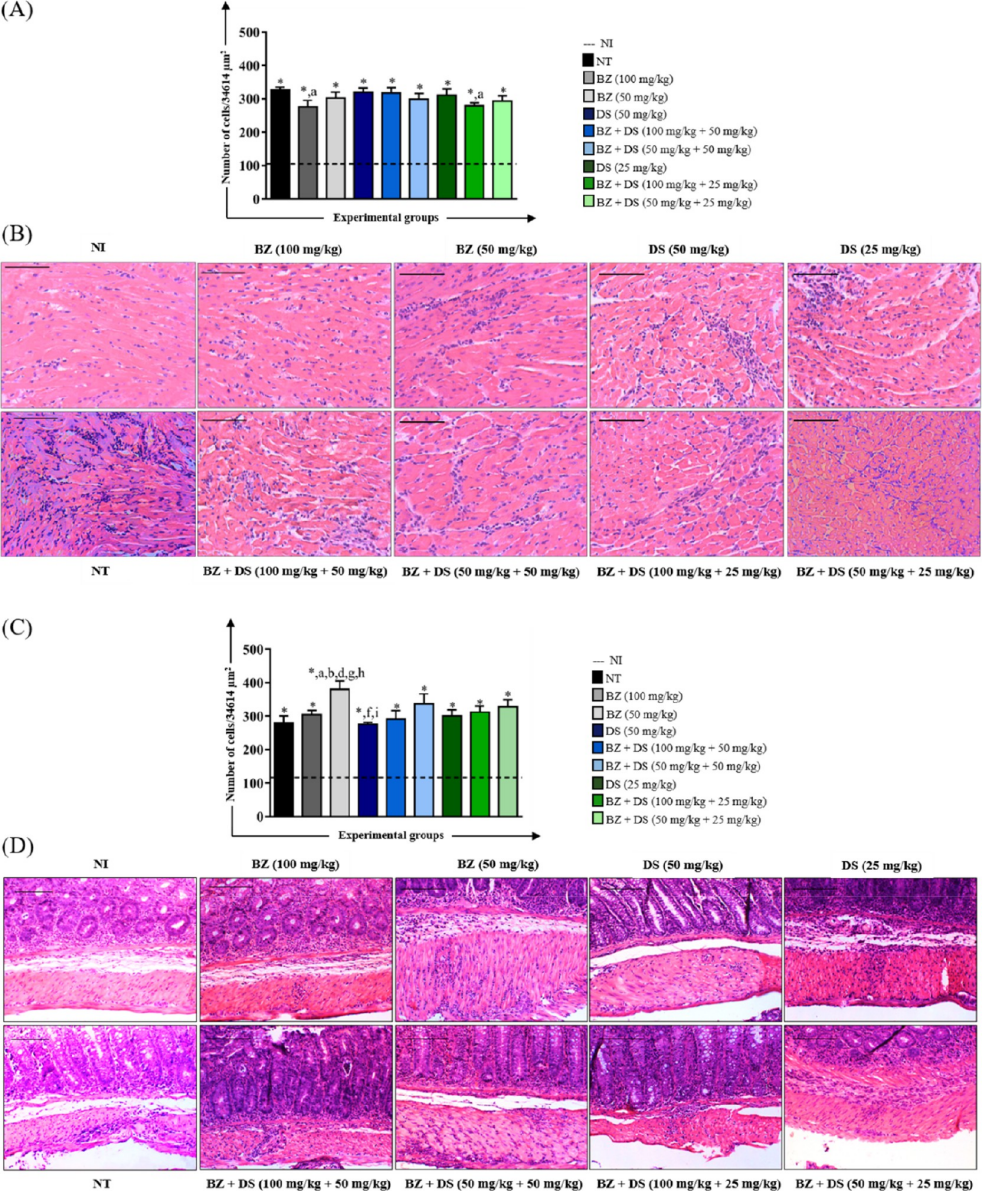
Morphometric analysis of the inflammatory process in the heart (A) and colon (C) and photomicrographs of histological sections of the heart (B) and colon (D) of mice infected with the VL-10 *T. cruzi* strain and treated in the chronic phase. * Number of inflammatory cells significantly higher (*p* < 0.05) than the uninfected group. The letters “a”, “b”, “d”, “f”, “g”, “h”, and “i” represent significant differences (*p* < 0.05) in relation to groups NT, BZ (100 mg/kg), DS (50 mg/kg), BZ + DS (50 mg/kg +50 mg/kg), DS (25 mg/kg), BZ + DS (100 mg/kg +25 mg/kg), and BZ + DS (50 mg/kg +25 mg/kg), respectively. The dashed line represents the average number of cell nuclei quantified in histological sections of uninfected animals. Values were expressed as mean ± standard error. Bar = 50 μm.

In the muscular layer of the colon, treatment with a lower dose of BZ resulted in a significantly higher number of inflammatory cells compared to several other treatment schemes (Figure 5C). In contrast, animals treated with DS alone (50 mg/kg) had a significantly lower number of inflammatory cells compared to groups treated with certain combinations of BZ + DS. Histological analysis demonstrated two intensities of the inflammatory infiltrate: intense in animals in the BZ (50 mg/kg) and BZ + DS (50 mg/kg +50 mg/kg) groups and moderate in animals in the other experimental groups (Figure 5D).

#### Fibrosis Analysis

Analysis of cardiac tissue from animals infected with the VL-10 *T. cruzi* strain and treated during the chronic phase showed that the area occupied by collagen fibers was greater in the groups treated only with DS or only with BZ at a dose of 50 mg/kg, with this fibrosis being of the same intensity as that observed in untreated animals (NT) (Figure 6A). However, animals treated with the combination of DS and BZ, at any dosage of DS, showed a significant reduction in the fibrosis area (Figure 6B). Among the combination regimens, those involving BZ (100 mg/kg) combined with DS (25 or 50 mg/kg) had the smallest fibrotic areas.

**Figure 6:**
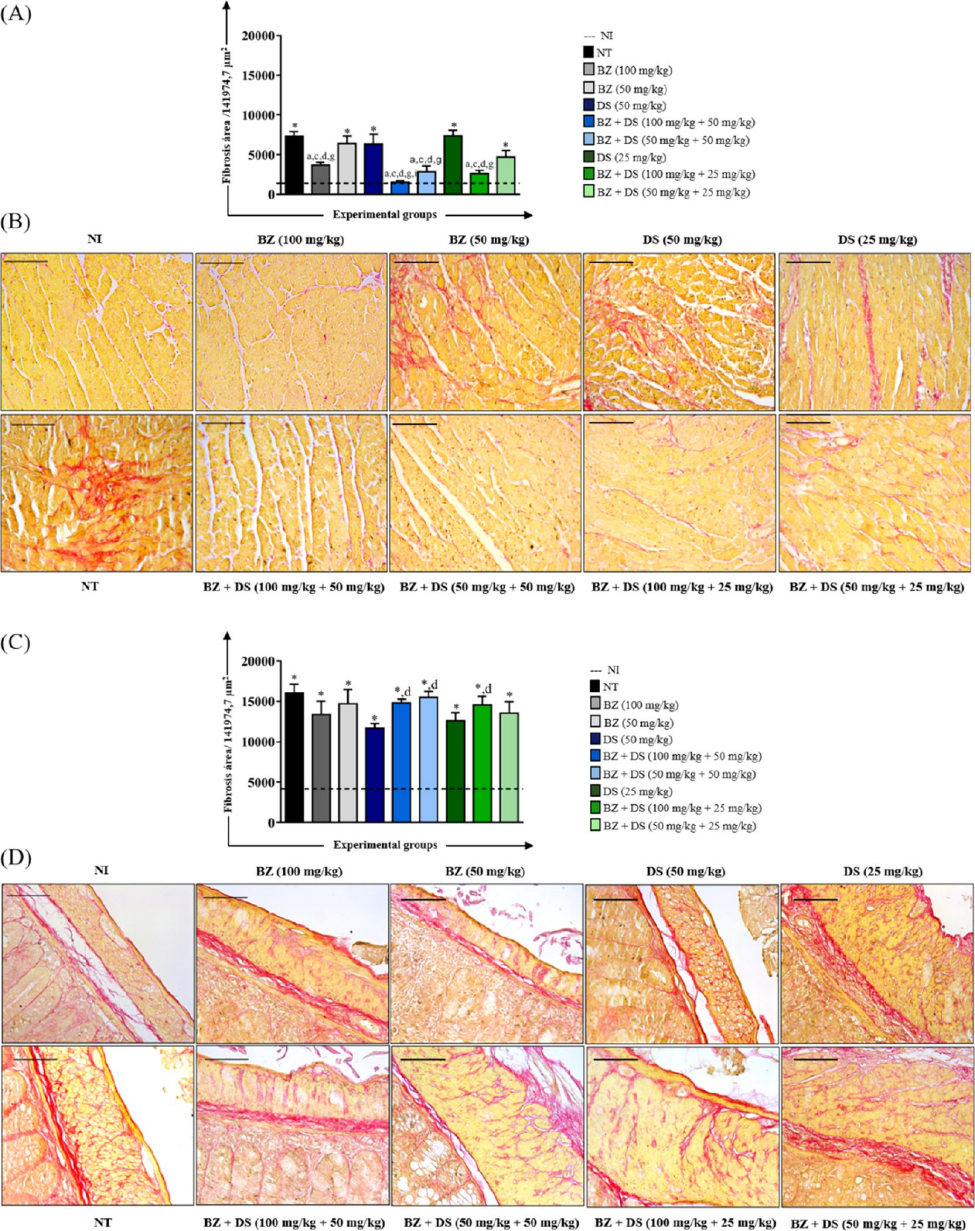
Morphometric analysis of the fibrosis area in the heart (A) and colon (C) and photomicrographs of histological sections of the heart (B) and colon (D) of mice infected with the VL-10 *T. cruzi* strain and treated in the chronic phase. *Area of fibrosis is significantly higher (*p* < 0.05) than the uninfected group. The letters “a”, “c”, “d”, “g”, and “i” represent significant differences (*p* < 0.05) in relation to NT, BZ (50 mg/kg), DS (50 mg/kg), DS (25 mg/kg), and BZ + DS (50 mg/kg +25 mg/kg) groups, respectively. The dashed line represents the mean of the fibrosis area quantified in histological sections of uninfected animals. Values were expressed as mean ± standard error. Bar = 50 μm.

Regarding the analysis of the colon muscle layer, the area occupied by collagen fibers in the NI group was significantly smaller than the area quantified in all other experimental groups (Figure 6C). The area of fibrosis in the DS group (50 mg/kg) was significantly smaller than the one quantified for 3 groups (BZ + DS 100 mg/kg +50 mg/kg, 50 mg/kg +50 mg/kg, and 100 mg/kg +25 mg/kg). Histological analysis showed that both the DS (50 mg/kg) and DS (25 mg/kg) groups presented discrete fibrosis areas (Figure 6D), ranging from moderate to intense in the animals belonging to the other experimental groups.

## Discussion

In this work, the evaluation of the DS trypanocidal activity in Swiss mice infected with the Y and VL-10 *T. cruzi* strains involved two strategies: drug repositioning and association. The DS repositioning is a promising approach since the safety and pharmacokinetic profiles of this drug have already been optimized for human use and manufacturing and storage factors have already been evaluated. In addition, the repositioning of drugs and combined therapies are relevant strategies in the search for faster and cheaper solutions.^38–40^ The association of drugs has been applied to several diseases and allows the use of at least two compounds that can act on different targets, contributing to the reduction of the risk of drug resistance, increasing therapeutic efficacy.^21,41^ In the search for new alternatives for the treatment of ChD, drug combination has been highlighted as a promising option in the literature.^20,21,42,43^

Almeida-Silva et al. (2022),^36^ for example, evaluated the effects of the association of BZ with DS and its derivative diethyldithiocarbamate against *T. cruzi* using both in vitro and in vivo approaches. Although both studies employed the Y strain, the inclusion of this strain in the present study provides independent and complementary data that contribute to a broader understanding of the effects and limitations of this drug combination while also reinforcing the relevance of standardized experimental models in this field.

Another important distinction between the studies is that the present work employed the VL-10 strain, whereas Almeida-Silva et al. (2022)^36^ used the Colombiana strain. Although both strains are classified as benznidazole resistant, they differ in relevant biological and pathological characteristics and belong to different discrete typing units (DTUs). The Colombiana strain is classified as DTU TcI, whereas the VL-10 strain belongs to DTU TcII. These DTUs are known to differ in the genetic profile, biological behavior, tissue tropism, and response to chemotherapy, factors that may influence disease progression and treatment outcomes.^44^ By including the VL-10 strain, our study aimed to extend the evaluation of the DS + BZ combination to a distinct genetic context, thereby assessing whether the therapeutic limitations and tissue-level effects observed are conserved across different benznidazole-resistant *T. cruzi* strains.

Another relevant point is that Almeida-Silva et al. (2022)^36^ reported a reduction in parasitemia in animals infected with the Y and Colombiana strains of *T. cruzi* following treatment with the BZ and DS combination, compared with low doses of BZ (20–50 mg/kg). In the present study, animals infected with the VL-10 strain of *T. cruzi* and treated with the different therapeutic regimens did not show a significant reduction in the area under the parasitemia curve when compared with animals treated with BZ (50–100 mg/kg). The differences in experimental outcomes between the studies may be explained, at least in part, by the fact that the strains used belong to different DTUs, as discussed above. Moreover, variability in experimental results is not unexpected in chemotherapy studies for *T. cruzi* and may arise from multiple methodological and biological factors.^45^

In this context, differences in experimental design, including drug doses and treatment duration, may have contributed to this variability as Almeida-Silva et al. (2022)^36^ used only suboptimal doses of benznidazole (10, 20, and 50 mg/kg) and treated animals for up to 60 days. Another important difference concerns drug solubilization: in the study by Almeida-Silva et al. (2022),^36^ the compounds were dissolved in dimethyl sulfoxide (DMSO) and stored at −20 °C until use, whereas in the present study, the drugs were suspended in 0.5% carboxymethylcellulose solution immediately before administration. In addition, subtle variations in animal handling, parasite passage history, and laboratory conditions are known to impact disease progression and treatment response.^14,46^

Regarding the dosage regimens evaluated in this study, BZ was administered at 100 mg/kg/day as this dose corresponds to the standard therapeutic regimen widely employed in experimental models of *T. cruzi* infection. In addition, a reduced dose of 50 mg/kg/day was included since this regimen has been explored by other research groups and has demonstrated biological and pharmacological relevance in vivo.^47–49^ DS dosing was established based on safety considerations and previous clinical and preclinical evidence. The maximum recommended safe dose of DS in humans is 500 mg/day; therefore, the doses evaluated in this study were selected to remain within the safety range reported in the literature. Furthermore, DS doses were chosen to be equal to or lower than the reduced BZ dose, allowing for comparative and combinatorial analyses while avoiding the exceedance of established toxicity thresholds.^50^

Quantitative PCR (qPCR) was used to evaluate the efficacy of DS, as well as two *T. cruzi* strains with varying levels of resistance to BZ and nifurtimox. It is known that therapeutic failure in ChD is defined by the persistence of the parasite that can be detected using different methods, such as FBE and PCR.^51^ Thus, qPCR is the recommended alternative to determine the parasite burden after treatment as well as parasitological cure.^52,53^

In this context, it is important to emphasize that the assessment of parasite load by qPCR represents a valuable complementary approach for evaluating therapeutic effects beyond parasitological cure. An limitation of the present study concerns the evaluation of parasite load as an additional indicator of the partial therapeutic efficacy. Parasite load determination in blood samples was restricted to animals that tested negative by FBE, and tissue parasite load (heart and colon) was assessed only in animals that were negative by blood qPCR. Future studies specifically designed to quantify parasite burden in all animals, regardless of parasitological status, will be essential to better characterize partial therapeutic effects beyond parasitological cure.

Overall, different parasitological cure profiles were observed, varying according to the *T. cruzi* strain, the type of treatment administered, and the evaluated organ. The highest parasitological cure rate obtained in the test carried out with the mice infected by the Y strain corroborates other authors, who have shown promising results from a series of associations between different classes of molecules and their activities in vitro and in vivo in different metabolic pathways of *T. cruzi*. The authors also pointed out that the use of drug combinations is a very promising strategy to increase therapeutic efficacy, decrease the inflammatory process, or improve the host’s immune response.^41^

This result can be explained by the fact that while BZ acts through reductive stress, involving covalent modification of macromolecules in nitroreduced intermediates, or by other nitroreduction interactions with the parasite components, DS interferes with proline metabolism, resulting in decreased parasite viability. The association tested in this work consists of drugs that have different mechanisms of action and synergistic effect, a feature that can hinder the development of resistance mechanisms by *T. cruzi*.

On the other hand, the concentration of TBARS and carbonylated protein was significantly higher in the groups treated with the associations, thus demonstrating that these in any of the tested concentrations of DS caused an intracellular increase in ROS (reactive oxygen species) and consequently an increase in lipid peroxidation and protein oxidation in these experimental groups. Thus, the association contributes to the increase in liver toxicity, since lipid peroxidation and protein oxidation cause loss of cellular functions through membrane degradation and inactivation of enzymes and cytoplasmic proteins. In the therapeutic schemes, associations with doses of 100 and 50 mg of BZ were used, and it was not possible with these doses to eliminate the toxicity of BZ. In this sense, studies of new therapeutic schemes with the objective of reducing liver damage caused by the association are necessary. It is important to emphasize that no animal in the untreated group or treated only with DS survived the end of the acute phase experiments with the Y *T. cruzi* strain; therefore, it was not possible to evaluate oxidative damage in these experimental groups.

Although no therapeutic success was observed in any of the experimental groups in animals infected with the VL-10 strain of *T. cruzi* during the acute phase of infection, experiments in the chronic phase were conducted to evaluate non curative therapeutic effects, such as reductions in inflammation or fibrosis. The results demonstrate that the VL-10 *T. cruzi* strain, in addition to being resistant to BZ, as already described in the literature, is also resistant to the treatment with DS. This diversity of therapeutic efficacy highlights the fact that *T. cruzi* is a species with heterogeneous genetic and phenotypic characteristics that have a major impact on pathogenesis, serodiagnosis, and drug discovery for ChD.^54,55^ Another relevant aspect is that several studies show that protozoans can differentiate in various ways throughout their life cycle, which could then explain their ability to develop drug resistance. *T. cruzi*, for example, can differentiate into dormant amastigotes, which could contribute to its persistence in the host’s tissue when treated with BZ.^56^

In addition, the results of parasitological healing are not NO-dependent. In general, regardless of the *T. cruzi* strain tested, higher concentrations of nitric oxide (NO) were observed in the plasma of animals treated in the acute phase of infection (Figures S1 and S2). This result was already expected since in the acute phase, macrophages infected by *T. cruzi* produce IL-12, a cytokine responsible for the initial synthesis of IFN-γ by NK cells, which activates macrophages that will produce high levels of NO, a trypanocidal molecule.^8,57^ However, in animals infected with the VL-10 *T. cruzi* strain and treated in the acute phase, even though high concentrations of NO were observed, no parasitological cure was seen in any of the experimental groups.

In this sense, it was also evaluated whether the treatment with DS would be able to improve the histopathological aspects, even though it did not promote a parasitological cure. This was determined through analysis of the inflammatory process and the presence of fibrosis in the cardiac muscle tissue and in the muscular layer of the colon. These organs were chosen for two reasons: (i) it is known that the intense inflammation of the myocardium (myocarditis) in combination with the persistence of the parasite, and that with the infiltration of lymphocytes, plasma cells, and macrophages, myocardial cells are also destroyed, forming “microabscesses” that later heal with fibrosis;^52,58^ (ii) gastrointestinal manifestations are in combination with high morbidity and are the second most common cause of complications in ChD. It is important to note that *T. cruzi* infection can affect all parts of the digestive system, but the esophagus and the colon are the most commonly affected organs.^59^ The inflammatory process can cause changes in the enteric nervous system and contribute to the development of pathological processes, including constipation and dysphagia.^60,61^

The results obtained through the histopathological evaluation presented different profiles that varied according to the treatment and organ. In experiments with the same strain, different responses to treatment were observed in early or already established infection stages, suggesting that different patterns of response to treatment may be related to the tissue distribution of parasites (time of infection), as well as the concentration of the drug in different tissues and not just genetic resistance, reinforcing the hypothesis of other researchers.^62,63^

Analysis of cardiac muscle tissue from animals infected with the Y *T. cruzi* strain demonstrated that combined therapy with BZ and DS, particularly at higher doses of DS, was more effective in reducing the inflammatory response compared with BZ monotherapy. This result corroborates with the one obtained in the analysis of tissue parasitism in animals treated with the combination of high doses of BZ + DS, in which no parasites were detected in the heart and colon of treated animals. Therefore, these results highlight the therapeutic potential of this combination since it significantly impairs the noninflammatory process in cardiac muscle tissue, allowed an increase in the parasitological cure rate when compared to the groups treated with BZ, and 100% animal survival. Thus, this result demonstrates that there is a direct relationship between parasitism and the number and extent of inflammatory foci in Chagasic myocarditis.

Quantitative analyses of the inflammatory process in cardiac muscle tissue and in the colonic muscle layer of animals infected with the VL-10 *T. cruzi* strain treated in both acute and chronic phases showed the best results in the analysis of the colon muscle layer. This result suggests that DS treatment may be effective in preventing neuronal destruction in this organ. This destruction is observed due to the presence of *T. cruzi*, which can affect the CNS and the peripheral nervous system (SNP).^60,64^ It is known that, due to inflammation, over time in the affected sites, both focal fibrosis and diffuse fibrosis develop, which is more important in the functional impairment of the affected organs. Due to this, in this study, the formation of fibrosis areas was also analyzed. The obtained results demonstrate that the association may play an important role in preventing the formation of fibrosis areas in the heart, while DS, at the highest tested concentration, was the one that stood out most in preventing the formation of fibrosis areas in the colon.

It is important to acknowledge that the histopathological analyses of cardiac and colonic tissues as well as the evaluation of hepatic oxidative stress markers were performed after the immunosuppression protocol. Although immunosuppression is a well-established and necessary approach to revealing residual parasitemia and to assess treatment failure in experimental ChD models, it may substantially modulate inflammatory responses and oxidative stress pathways. Therefore, the inflammatory and fibrotic profiles observed in this study may not fully reflect the steady-state tissue condition and could have been influenced, at least in part, by the immunosuppressive regimen. Nevertheless, all experimental groups were subjected to the same immunosuppression protocol, allowing for consistent comparative analyses among treatments.^65–67^

Although in vitro tests have shown that DS has trypanocidal activity,^33^ in this study, both groups infected either with the Y or VL-10 *T. cruzi* strains and treated only with DS at the two tested concentrations did not show entirely promising results. Variations between in vitro vs in vivo results are frequently observed in the literature, but the understanding of the mechanisms underlying these differences is still limited. However, it is important to note that the activity of the drug in vivo depends on specific biological processes within the organism, which are not simulated in vitro, such as bioavailability, absorption, gastrointestinal transit, pH change, and permeability in the gastrointestinal tract.^68–70^

Variations were also observed among the results obtained for the different strains tested. Although both Y and VL-10 strains belong to the same DTU (TcII), they display marked biological heterogeneity, including differences in virulence, tissue tropism, parasitemia profiles, and susceptibility to BZ, which may account for the distinct therapeutic responses observed in this study.^44,71^

In addition, the disagreement in the results demonstrates that the development of new drugs that are effective against *T. cruzi* is surrounded by major challenges marked by the difficulty of standardizing in vivo and in vitro tests for the screening of new compounds.^72^ ChD models involve several parameters that are often uncontrolled, including the relationship between exposure and cure, route of administration, time and duration of treatment, strain of mice, interactions between host-parasite, and type of immune response, in addition to other challenges, such as definitions of cure criteria and stage of the disease. These factors can affect treatment outcomes and, therefore, attributing therapeutic failure only to the lack of susceptibility of *T. cruzi* to the tested compounds should be considered carefully.^73,74^

## Conclusion

Although the combination of DS and BZ at different concentrations has shown some benefit, DS alone is not entirely effective. In this perspective, more studies are necessary to investigate other therapeutic strategies, such as decreasing drug concentrations with increased treatment time duration and the frequency of administration, which could be a promising alternative. Undoubtedly, maintaining the baseline levels of drugs in the plasma without causing toxicity would be a necessary strategy to achieve therapeutic success.

## Materials and Methods

### Ethics Statement

All procedures were carried out in accordance with the ethical principles recommended by Conselho Nacional de Controle de Experimentação Animal (CONCEA), having previously been approved by the Ethics Committee on the Use of Animals (CEUA) of the Federal University of Ouro Preto (Protocol n. 8572120418). Clinical trial number: not applicable.

### Animals and Experimental Groups

300 female Swiss mice (18–25 g), born at the maternity ward in the Animal Facility at Federal University of Ouro Preto (CCA-UFOP) were used for this study. The animals were then acclimated at CCA-UFOP for a period of 7 days prior to the beginning of the experiments. Five animals were housed per box in a ventilated rack under controlled conditions of light (12/12 h, light/dark), temperature (23 ± 2 °C), and relative humidity (70%). They were provided with autoclaved water and Nuvilab commercial food ad libitum.

The animals were further divided into three main experimental groups (*n* = 100/group): infected with the Y *T. cruzi* strain and treated in the acute phase; infected with the VL-10 *T. cruzi* strain and treated in the acute phase; and infected with the VL-10 *T. cruzi* strain and treated in the chronic phase of infection. Each experimental group was divided into 10 subgroups according to treatment (*n* = 10/subgroup): untreated (NT); BZ (100 mg/kg); BZ (50 mg/kg); DS (50 mg/kg); DS (25 mg/kg); BZ + DS (100 mg/kg +50 mg/kg); BZ + DS (50 mg/kg +50 mg/kg); BZ + DS (100 mg/kg +25 mg/kg); and BZ + DS (50 mg/kg +25 mg/kg). A group of uninfected and untreated animals (NI) was also used as a control.

### Evaluation of Swiss Mice Infected with the Y or VL-10 *T. cruzi* Strains and Treated with Disulfiram in the Acute Phase

The infection was performed intraperitoneally, with 1 × 10^4^ trypomastigotes forms of Y or VL-10 *T. cruzi* strains. Only animals that tested positive for parasitemia were included in the study,^75^ and treatment started on the same day that the infection was confirmed. Thirty days after the end of treatment, the immunosuppression protocol was initiated, and the animals were euthanized about 6 days after the end of the immunosuppression protocol. The following samples were collected from them: blood to assess the parasite load, serum for NO measurements, and heart and colon for inflammatory process analysis. Liver samples were also collected for oxidative stress marker analysis in order to assess liver damage under the different therapeutic schemes tested.

### Evaluation of Swiss Mice Infected with the VL-10 *T. cruzi* Strain and Treated with Disulfiram in the Chronic Phase

#### Infection

The infection was performed intraperitoneally, with 5 × 10^3^ trypomastigotes forms of VL-10 *T. cruzi* strains. Only animals that showed positive parasitemia in previous tests were included in the study, and a parasitemia was plotted.^76^ Treatment started 120 days after the infection was confirmed. Thirty days after the end of treatment, the immunosuppression protocol was initiated. Approximately 6 days after the end of the immunosuppression protocol, the animals were euthanized. Blood was collected to assess the parasite load, serum was collected for the measurement of NO, and both the heart and colon were also sampled for analysis of the inflammatory process and collagen neoformation.

#### Parasitemia

The FBE was performed to confirm the infection of animals in all experimental groups. Summing up, parasites were counted in 50 microscopic fields of a wet preparation containing 5 μL of blood collected from the mice’s caudal vein. Microscopic blood parasite examinations were performed daily until no parasites were observed for five consecutive days, and the results were expressed as parasites/0.1 mL of blood.^76^

#### Survival Rate

The animals (the same ones used to determine parasitemia) were observed daily until the day of euthanasia. Survival was recorded and expressed as a cumulative percentage.

#### Treatment

DS (MP Biomedicals, LLC) and BZ (LAFEPE, Recife, Brazil) were suspended in a 0.5% carboxymethylcellulose solution (Sigma-Aldrich, St. Louis, MO, USA), according to the tested concentrations. Subsequently, they were administered orally (gavage) in a single daily dose for 20 days. The animals in the control group received 200 μL of water via gavage.

#### Immunosuppression

Immunosuppression was performed 30 days after the end of treatment using the drug cyclophosphamide, *N*,*N*-bis (2-chloroethyl)-1,3,2-oxazaphosphinan-2-amine-2-oxide, (Genuxal, Baxter, Deerfield, Illinois, USA). It consisted of three cycles of 50 mg/kg/day, for 4 consecutive days, with an interval of 3 days between each cycle. FBE was performed for 5 days after the end of immunosuppression to check the animals’ parasitemia levels.^75^

#### Euthanasia

Approximately 6 days after the end of the immunosuppression protocol, the animals were euthanized. Sedation was carried out intraperitoneally using ketamine (Syntec, São Paulo, Brazil; 90 mg/kg) and xylazine (Ceva, São Paulo, Brazil) (9 mg/kg). After complete sedation and anesthesia, 100 μL of blood for qPCR and approximately 400 μL of blood for serum were collected from the orbital plexus using a Pasteur pipet. The animals were euthanized by cervical dislocation, and the heart and colon were then collected for histopathological evaluations.

#### Quantitative Polymerase Chain Reaction

Initially, genomic DNA was extracted from whole blood (100 μL), heart, and colon (15–25 mg) samples using the DNeasy Blood & Tissue Kit (Qiagen, Hilden, Germany) and Wizard (Promega, Madison, Wisconsin, USA), respectively, as instructed by the manufacturers. The concentration and purity of the extracted DNA were determined using a nanospectrophotometer (NanoDrop 2000, Thermo Scientific, USA) at 260/280 and 260/230 nm wavelengths. For the qPCR reaction, the following primers were used with *T. cruzi* DNA as a template: TCZ—F 5′-GCTCTTGCCCACAMGGGTGC-3′, where M = A or C, and TCZ—R 5′-CCAAGCAGCGGATAGTTCAGG-3′, resulting in the amplification of a 132bp-long fragment (Cummings and Tarleton 2003). The samples used for the standard curve were considered positive controls, whereas wells containing only nuclease-free water (without DNA) were considered the negative controls as well as the NI group samples that were subjected to the reaction with the specific *T. cruzi* primers to control nonspecific reaction. Amplification of the murine tumor specific necrosis factor alpha (TNF-α) gene was performed to verify the integrity of the analyzed DNA. The reactions were prepared as follows: 60 ng/μL genomic DNA, 5 μL of Go Taq qPCR Master Mix (Promega), 2 μL of primers, and nuclease-free water to a final volume of 10 μL per well. The qPCR reactions were performed in duplicate, using 96-well plates—MicroAmpOptical 96—Well Reaction Plate (Applied Biosystems by Life Technologies, USA), sealed with optical adhesives—Optical Adhesive Covers (Applied Biosystems by Life Technologies, USA) in an ABI Prism 7500 Sequence Detection System (Applied Biosystems, USA) thermal cycler.

#### Histopathological Assessments

The tissues were fixed in 10% buffered formaldehyde solution (pH 7.2), embedded in paraffin, and subjected to microtomy to obtain sections with a thickness of 4 μm. Subsequently, the sections were stained with hematoxylin and eosin (HE) to quantify the inflammatory process in 20 random images (40× objective) or Picrosirius Red to quantify the neoformation of collagen in 10 random images (20× objective). The images were digitalized using a Leica DM5000B microscope coupled to a DFC300FX camera. The analyses were performed by using Leica QwinV3 software. Photodocumentation and morphometry were performed at the MultiUser Laboratory of the Biological Sciences Research Center at the Federal University of Ouro Preto.

#### Quantification of Oxidative Damage in the Liver

To evaluate the oxidative damage in the liver, dosage of TBARS and measurement of carbonyl protein were executed according to the methodologies described by Buege and Aust (1978)^77^ and Levine et al. (1994),^78^ respectively.

### Statistical Analysis

The statistical tests were conducted by GraphPad Prism 5.0 software (Prism Software, Irvine, CA, USA), with the differences being considered significant when *p* values were less than 0.05 (*p* < 0.05). The D’Agostino-Pearson test was used to assess data normality. For data classified as parametric, analysis of variance (one-way ANOVA) and Student’s *t* test were performed to compare between groups. Nonparametric data were analyzed using the Mann-Whiteny or Kruskal–Wallis followed Dunns test.

## Supplementary Material



## Notes

Dosage of NO in the serum of animals infected with the Y *T. cruzi* strain and treated in the acute phase and dosage of NO in the serum of animals infected with the VL-10 *T. cruzi* strain and treated in the acute/chronic phase (PDF)

## Abbreviations

CCA-UFOP: Animal Facility at Federal University of Ouro Preto
BZ: benznidazole
CNS: central nervous system
ChD: Chagas disease
CONCEA: Conselho Nacional de Controle de Experimentação Animal
TcP5CDH: delta-1-pyrroline-5-carboxylate dehydrogenase
DETC: diethyldithiocarbamate
DS: disulfiram
ECG: electrocardiogram
CEUA: ethics committee on the use of animals
UFOP: Federal University of Ouro Preto
HE: hematoxylin and eosin
TNF-α: necrosis factor alpha
NO: nitric oxide
NI: noninfected
SNP: peripheral nervous system
qPCR: quantitative polymerase chain reaction
ROS: reactive oxygen species
FBE: fresh blood examination
TBARS: thiobarbituric acid reactive substances
*T. cruzi*: *Trypanosoma cruzi*
NT: untreated
