# Supplementary material for: In Vivo Trypanocidal Activity of the Drug Disulfiram Combined with Benznidazole
Source: ACS Infect Dis. 2026 Mar 31;12(4):1313–28. doi: 10.1021/acsinfecdis.5c00936 (PMC13077684; doi:10.1021/acsinfecdis.5c00936)
Supplement: Supplementary file 1 [file id5c00936_si_001.pdf]

Supporting Information

In vivo trypanocidal activity of the drug Disulfiram combined with Benznidazole

Viviane Flores Xavier[1], *Kátia da Silva Fonseca*[2], *Thays Helena Chaves Duarte*[1], *Flávia de Souza Marques*[1], *Lucas Resende Dutra Sousa*[1], *Aline Tonhela Ferraz*[1], *Fernanda Caetano Camini*[3], *Daniela Caldeira Costa Calsavara*[3], *Cláudia Martins Carneiro*[2], *Brian Alejandro Suárez Mantilla*[4], *Ariel Mariano Silber*[4], *Paula Melo de Abreu Vieira*[1*]

[1] Laboratory of Morphopathology, Department of Biological Sciences, Nucleus of Biological Sciences Research, Institute of Exact and Biological Sciences, Federal University of Ouro Preto, Ouro Preto, Minas Gerais, Brazil.

[2] Laboratory of Immunopathology, Nucleus of Biological Sciences Research, Institute of Exact and Biological Sciences, Federal University of Ouro Preto, Ouro Preto, Minas Gerais, Brazil.

[3] Laboratory of Biochemistry Metabolic, Department of Biological Sciences, Institute of Exact and Biological Sciences, Federal University of Ouro Preto, Ouro Preto, Minas Gerais, Brazil.

[4] Laboratory Biochemistry of Tryps, Department of Parasitology, Institute of Biomedical Sciences, University of São Paulo, São Paulo, Brazil.

* Corresponding Author

paula@ufop.edu.br

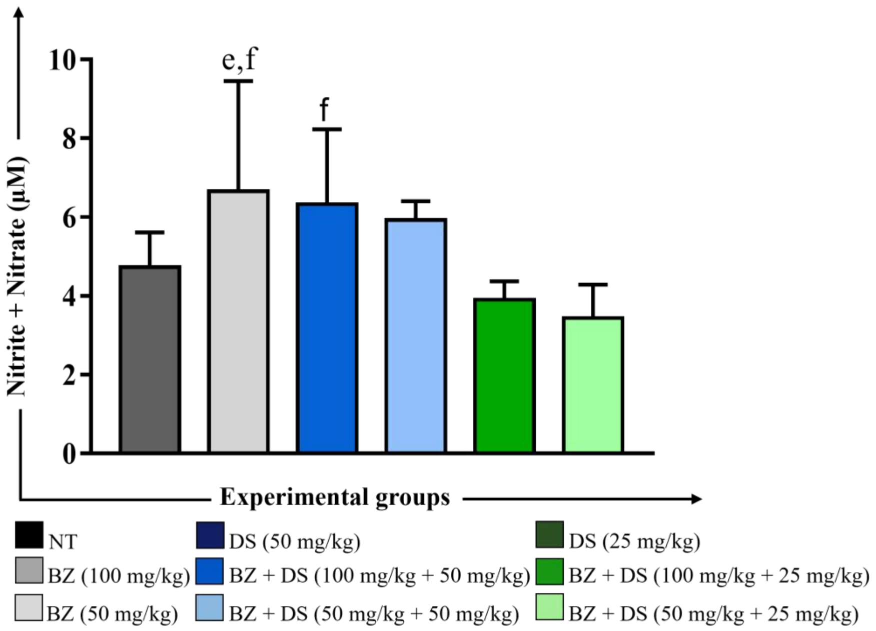

Fig. S1 Dosage of nitric oxide in the serum of animals infected with the Y strain of *T. cruzi* and treated in the acute phase. The letters "e" and "f" represent significant differences ($p < 0.05$) in relation to the BZ + DS (100 mg/kg + 25 mg/kg) and BZ + DS (50 mg/kg + 25 mg/kg) groups, respectively. Values were expressed as mean ± standard error.

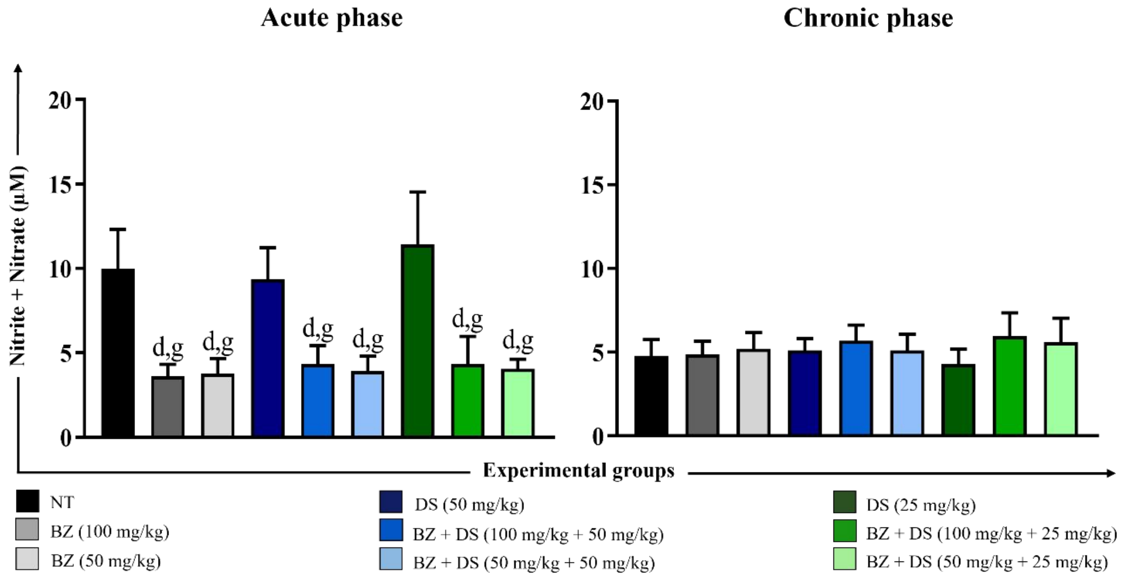

Fig. S2 Dosage of nitric oxide in the serum of animals infected with the VL-10 strain of *T. cruzi* and treated in the acute/chronic phase. The letters "d" and "g" represent significant differences ($p < 0.05$) in relation to DS (50 mg/kg) and DS (25 mg/kg) groups, respectively. Values were expressed as mean ± standard error.
